# Supplementary material for: Organization of circuits linking descending input to motor output in the Drosophila Male Adult Nerve Cord connectome
Source: eLife. 2026 Jul 20;13:RP96084. doi: 10.7554/eLife.96084 (PMC13384506; doi:10.7554/eLife.96084)
Supplement: Peer review history

# Editor report: eLife Assessment

**DOI:** 10.7554/eLife.96084.3.sa0

**Authors:** Mani Ramaswami

**Affiliations:** Trinity College Dublin Ireland

**Keywords:** Important, Solid

This paper describes the structure and connectivity of brain neurons that send descending connections to motor neurons and muscle in the fruit fly nerve cord, using a synapse-resolution connectome. This **important** work provides a wealth of hypotheses and predictions for future experimentation and modelling. Using state-of-the-art methods, the authors provide **solid** evidence for their conclusions.

# Referee report: Reviewer #1 (Public review)

**DOI:** 10.7554/eLife.96084.3.sa1

Summary:

Cheong et al. use a synapse-resolution wiring map of the fruit fly nerve cord to comprehensively investigate circuitry between descending neurons (DNs) from the brain and motor neurons (MNs) that enact different behaviours. These neurons were painstakingly identified, categorised, and linked to existing genetic driver lines; this allows the investigation of circuitry to be informed by the extensive literature on how flights walk, fly, and escape from looming stimuli. New motifs and hypotheses of circuit function were presented. This work will be a lasting resource for those studying nerve cord function.

Strengths:

The authors present an impressive amount of work in reconstructing and categorising the neurons in the DN to MN pathways. There is always a strong link between the circuitry identified and what is known in the literature, making this an excellent resource for those interested in connectomics analysis or experimental circuits neuroscience. Because of this, there are many testable hypotheses presented with clear predictions, which I expect will result in many follow-up publications. Most MNs were mapped to the individual muscles that they innervate by linking this connectome to pre-existing light microscopy datasets. When combined with past fly brain connectome datasets (Hemibrain, FAFB) or future ones, there is now a tantalising possibility of following neural pathways from sensory inputs to motor neurons and muscle.

Weaknesses:

As with all connectome datasets, the sample size is low, limiting statistical analyses. Readers should keep this in mind, but note that this is the current state-of-the-art. Some figures are weakened by relying too much on depictions of wiring diagrams without additional quantification of connectivity. Readers may find the length of this work challenging, particularly the initial anatomical descriptions of the dataset, which span many figures and may not be of interest to those outside of the subfield.

# Referee report: Reviewer #2 (Public review)

**DOI:** 10.7554/eLife.96084.3.sa2

Summary:

In Cheong et al., the authors analyze a new motor system (ventral nerve cord) connectome of *Drosophila*. Through proofreading, cross-referencing with another female VNC connectome, they define key features of VNC circuits with a focus on descending neurons (DNs), motor neurons (MNs), and local interneuron circuits. They define DN tracts, MNs for limb and wing control and their nerves (although their sample suffers for a subset of MNs). They establish connectivity between DNs and MNs (minimal). They perform topological analysis of all VNC neurons including interneurons. They focus specifically on identifying core features of flight circuits (control of wings and halteres), leg control circuits with a focus on walking rather than other limbed behaviors (grooming, reaching, etc.), intermediate circuits like those for escape (GF). They put these features in the context of what is known or has been posited about these various circuits.

Strengths

Some strengths of the manuscript include the matching of new DN and MN types to light microscopy, including serial homology of leg motor neurons. This is a valuable contribution that will certainly open up future lines of experimental work. As well, the analysis of conserved connectivity patterns within each leg neuromere and interconnecting connectivity patterns between neuromeres will be incredibly valuable. The standard leg connectome is very nice. Finally, the finding of different connectivity statistics (degrees of feedback) in different neuropils is quite interesting and will stimulate future work aimed at determining its functional significance.

Weaknesses

The degradation of many motor neurons is unfortunate. Figure 5 supplement 1 shows that roughly 50% of the leg motor neurons have significantly compromised connectivity data, whereas for non-leg motor neurons, few seem to be compromised. As well, the infomap communities don't seem to be so well controlled/justified. Community detection can be run on any graph - why should I believe that the VNC graph is actually composed of discrete communities? Perhaps this comes from a lack of familiarity with the infomap algorithm, but I imagine most readers will be similarly unfamiliar with it, so more work should be done to demonstrate the degree to which these communities are really communities that connect more within than across communities.

# Author comment: Author response

**DOI:** 10.7554/eLife.96084.3.sa3

**Authors:** Han SJ Cheong, Katharina Eichler, Tomke Stürner, Samuel K Asinof, Andrew S Champion, Elizabeth C Marin, Tess Oram, Marissa Sumathipala, Lalanti Venkatasubramanian, Shigehiro Namiki, Igor Siwanowicz, Marta Costa, Stuart Berg, Janelia FlyEM Project Team, Gregory SXE Jefferis, Gwyneth M Card

**Affiliations:** Columbia University New York United States; Leipzig University Cambridge United Kingdom; MRC Laboratory of Molecular Biology Cambridge United Kingdom; Janelia Research Campus Ashburn United States; University of Cambridge Cambridge United Kingdom; University of Cambridge Cambridge United Kingdom; Janelia Research Campus Ashburn United States; Janelia Research Campus Ashburn United States; University of Cambridge Cambridge United Kingdom; Janelia Research Campus Ashburn United States; Janelia Research Campus Ashburn United States; University of Cambridge Cambridge United Kingdom; Janelia Research Campus, Howard Hughes Medical Institute Ashburn United States; Janelia Research Campus Ashburn United States; MRC Laboratory of Molecular Biology Cambridge United Kingdom; Howard Hughes Medical Institute New York United States

The following is the authors’ response to the original reviews.

**Reviewer #1 (Public Review):**

Summary:

Cheong et al. use a synapse-resolution wiring map of the fruit fly nerve cord to comprehensively investigate circuitry between descending neurons (DNs) from the brain and motor neurons (MNs) that enact different behaviours. These neurons were painstakingly identified, categorised, and linked to existing genetic driver lines; this allows the investigation of circuitry to be informed by the extensive literature on how flights walk, fly, and escape from looming stimuli. New motifs and hypotheses of circuit function were presented. This work will be a lasting resource for those studying nerve cord function.

Strengths:

The authors present an impressive amount of work in reconstructing and categorising the neurons in the DN to MN pathways. There is always a strong link between the circuitry identified and what is known in the literature, making this an excellent resource for those interested in connectomics analysis or experimental circuits neuroscience. Because of this, there are many testable hypotheses presented with clear predictions, which I expect will result in many follow-up publications. Most MNs were mapped to the individual muscles that they innervate by linking this connectome to pre-existing light microscopy datasets. When combined with past fly brain connectome datasets (Hemibrain, FAFB) or future ones, there is now a tantalising possibility of following neural pathways from sensory inputs to motor neurons and muscle.

Weaknesses:

As with all connectome datasets, the sample size is low, limiting statistical analyses. Readers should keep this in mind, but note that this is the current state-of-the-art. Some figures are weakened by relying too much on depictions of wiring diagrams as evidence of circuit function, similarity between neuropils, etc. without additional quantitative justification.

We thank the reviewer for their helpful comments. We are excited about the release of this densely reconstructed connectome and its potential to facilitate circuit exploration in the VNC. We note that while statistical methods for analyzing complicated networks such as the connectome are still being developed, the wiring diagrams presented are themselves visualizations of quantitative data. We address specific concerns below.

**Reviewer #2 (Public Review):**

Summary:

In Cheong et al., the authors analyze a new motor system (ventral nerve cord) connectome of *Drosophila*. Through proofreading, cross-referencing with another female VNC connectome, they define key features of VNC circuits with a focus on descending neurons (DNs), motor neurons (MNs), and local interneuron circuits. They define DN tracts, MNs for limb and wing control, and their nerves (although their sample suffers for a subset of MNs). They establish connectivity between DNs and MNs (minimal). They perform topological analysis of all VNC neurons including interneurons. They focus specifically on identifying core features of flight circuits (control of wings and halteres), leg control circuits with a focus on walking rather than other limbed behaviors (grooming, reaching, etc.), and intermediate circuits like those for escape (GF). They put these features in the context of what is known or has been posited about these various circuits.

Strengths:

Some strengths of the manuscript include the matching of new DN and MN types to light microscopy, including the serial homology of leg motor neurons. This is a valuable contribution that will certainly open up future lines of experimental work.

Also, the analysis of conserved connectivity patterns within each leg neuromere and interconnecting connectivity patterns between neuromeres will be incredibly valuable. The standard leg connectome is very nice.

Finally, the finding of different connectivity statistics (degrees of feedback) in different neuropils is quite interesting and will stimulate future work aimed at determining its functional significance.

We thank the reviewer for their constructive feedback, and are optimistic about the utility of the MANC connectome to the *Drosophila* neurobiology community in dissecting VNC circuit function.

Weaknesses:

First, it seems like quite a limitation that the neurotransmitter predictions were based on training data from a fairly small set of cells, none of which were DNs. It's wonderful that the authors did the experimental work to map DN neurotransmitter identity using FISH, and great that the predictions were overall decently accurate for both ACh and Glu, but unfortunate that they were not accurate for GABA. I hope there are plans to retrain the neurotransmitter predictions using all of this additional ground truth experimental data that the authors collected for DNs, in order to provide more accurate neurotransmitter type predictions across more cell types.

The reviewer makes an excellent suggestion, and collecting further ground truth data and retraining the neurotransmitter classifier is an ongoing research project.

Second, the degradation of many motor neurons is unfortunate. Figure 5 Supplement 1 shows that roughly 50% of the leg motor neurons have significantly compromised connectivity data, whereas, for non-leg motor neurons, few seem to be compromised. If that is the correct interpretation of this figure, perhaps a sentence like this that includes some percentages (~50% of leg MNs, ~5% of other MNs) could be added to the main text so that readers can get a sense of the impact more easily.

Thank you for this suggestion. We have added a line describing the percentage of leg and other MNs affected (L416-417).

As well, Figure 5 Supplement 1 caption says "Note that MN groups where all members of the group have reconstruction issues may not be flagged" - could the authors comment on how common they think this is based on manual inspection? If it changes the estimate of the percentage of affected leg motor neurons from 50% to 75% for example, this caveat in the current analysis would need to be addressed more directly. Comparing with FANC motor neurons could perhaps be an alternative/additional approach for estimating the number of motor neurons that are compromised.

We agree that a direct comparison to another dataset, such as FANC, would aid in identifying reconstruction issues. However, a full analysis is not currently possible as only a minority of FANC neurons have been proofread or annotated. We were able to gain some insights into reconstruction quality by looking at T1 motor neurons, where FANC MN reconstruction is more complete. As reported in the submitted manuscript, we were able to confidently match T1 MNs between FANC and MANC for all but one MN (we are missing one ltm MN on the right side of MANC). While some of the MANC neurons had smaller/less dense arbors than FANC, none of them would have been flagged as having reconstruction issues. However, for FANC, we observe that neurons on the right have less dense arbors and fewer reconstructed synapses than neurons on the left. We have prepared a reviewer figure analyzing the consistency of synapse counts for the T1 (front leg) MNs:

**Author response image 1.** 

In these results (MANC on the left, FANC on the right) we compare the number of input synapses on matched motor neurons on the left (LHS) and right hand side (RHS) of each dataset. We see that the MANC distribution is much more symmetric, indicating left and right hand side synapse counts for matched MNs are more similar in MANC. This is likely largely due to the left-right difference in reconstruction completeness in the FANC T1 leg neuropils. The number of synapses per cell type is also more variable in FANC. Overall, we recommend that end users should inspect the morphology and total synapse counts of individual MNs of interest in either dataset as part of any detailed analysis.

This analysis might benefit from some sort of control for true biological variability in the number of MN synapses between left and right or across segments. I assume the authors chose the threshold of 0.7 because it seemed to do a good job of separating degraded neurons from differences in counts that could just be due to biological variability or reconstruction imperfections, but perhaps there's some way to show this more explicitly. For example, perhaps show how much variability there is in synapse counts across all homologs for one or two specific MN types that are not degraded and are reconstructed extremely well, so any variability in input counts for those neurons is likely to be biologically real. Especially because the identification of serial homologs among motor neurons is a key new contribution of this paper, a more in-depth analysis of similarities and differences in homologous leg MNs across segments could be interesting to the field if the degradation doesn't preclude it.

We agree that there can be ambiguity in whether variability in synapse counts between left-right homologs of a MN type represents biological variability or technical issues. We have added a comparison of synapse counts of T1 leg MNs in MANC (Left) vs FANC (Right) as noted in the previous point. As the number of connectomes available to us increases, we will have a better idea of how synapse counts of MNs vary within and between animals.

Fourth, the infomap communities don't seem to be so well controlled/justified. Community detection can be run on any graph - why should I believe that the VNC graph is actually composed of discrete communities? Perhaps this comes from a lack of familiarity with the infomap algorithm, but I imagine most readers will be similarly unfamiliar with it, so more work should be done to demonstrate the degree to which these communities are really communities that connect more within than across communities.

A priori we expect that there is some degree of functional division between circuits controlling different limbs or motor systems, given current evidence that VNC neuropils and neural hemilineages are relatively specialized in controlling motor output. We have added this explanation to section 2.4.2 (L633-635).

The Infomap algorithm was chosen out of several directed and undirected community detection methods that we tried, as it defined communities that each had connectivity with narrow and specific motor neuron subclasses. For example, it labeled populations in each of the six leg neuropils as belonging to distinct communities. We think this provides an interesting partitioning of the VNC network that could have biological relevance (which future functional studies should investigate). To the reviewer’s final sentence, we do show intra- vs inter-community connectivity in Fig. 9–supplement 1B. Notably, most communities except several small ones have far more intra-community connectivity than inter-community connectivity. We have added text highlighting this observation (L656-658).

We do, however, agree with the general point of the reviewer that it is not yet known which community detection methods are ‘optimal’ for use with connectomics data, so we have added further text (L679-683) explaining that community detection in MANC will require further investigation and validation in the future.

I think the length of this manuscript reduces its potential for impact, as I suspect the reality is that many people won't read through all 140 pages and 21 main figures of (overall excellent) work and analysis.

We intend this paper to serve not only as a first look into the organization of descending-to-motor circuits, but also as a resource for future investigations in MANC. The provided detail is intended to serve these purposes.

**Reviewer #1 (Recommendations For The Authors):**

General comments:

I find that there are too many main figures with too much content in them, as well as too much corresponding text. Much of the initial anatomical identification and description could be summarised in fewer main figures, with more supplementary figures if the authors desired. I think there is a lot of great insight in this paper, particularly in the second half, but I am concerned that the extensive detail in the initial sections may challenge reader engagement through to the later sections of the paper. It would also be useful to have a higher level and shorter discussion.

Reiterating our response from above, we intend this paper to serve not only as a first look into the organization of descending-to-motor circuits, but also as a resource for future investigations in MANC. The provided detail is intended to serve these purposes.

There is sometimes an over-reliance on wiring diagrams or complex plots as evidence without further quantification. I will mention several examples below, as well as additional suggestions.

Specific comments:

In Figure 2E, how are DNs divided into pair vs population type? This was a very interesting idea, particularly in light of "command-like" neurons vs ensembles of DNs controlling behaviour. However, it is not clear how this distinction is made. This concept is referenced throughout the manuscript, so I think a clear quantitative way of identifying "pair" vs "population" identity for each DN would be very useful. And at the very least, a thorough explanation of how it is done in the current manuscript.

We have added additional text in the Figure 2 legend to point towards Materials and Methods where the DN grouping (pair vs. population) is explained. These groups were formed based on morphology and further split into types based on connectivity, if needed. However, as the connectome represents a static snapshot of connectivity with no functional data, it remains possible that some DNs that were grouped as populations may act functionally as multiple pairs. Future work should continue to update these annotations.

In Figure 4, there are some inconsistencies between neurotransmitter predictions and experimental FISH data. Have the authors taken into consideration Lacin et al. 2019 (https://elifesciences.org/articles/43701)? Specifically in that paper, it is stated: "We did not find any cases of neurons using more than one neurotransmitter, but found that the acetylcholine specific gene ChAT is transcribed in many glutamatergic and GABAergic neurons, but these transcripts typically do not leave the nucleus and are not translated." I wonder if this might explain some of the inconsistencies between FISH (mRNA detection) and the neurotransmitter predictions (presumably based on indirect protein structures detected via EM imagery), or the presence of so much co-transmission.

We agree and have added this possible explanation for apparent co-transmission in the text (L394-397).

In Figure 8B, the authors state: "We found that individual DN and MN subclasses have direct downstream and upstream partners, respectively, that are relatively hemilineage-restricted (Figure 8B)." While the connectivity patterns highlighted are intriguing, further quantitative analysis could help strengthen this point. The connectivity matrices in Figure 8B are linked to activation phenotypes and hemilineages below. But I don't really know how to interpret "relatively hemilineage-restricted" in light of this plot. How does this connectivity pattern for example compare statistically to a randomly selected set of DNs (maintaining the same group size for example)? Would random DN sets be less hemilineage restricted? Similar quantification would be helpful to support this statement "...with high correspondence between the hemilineages connected to individual DN and MN subclasses that are expected to be functionally related."

"both upper tectulum DNs (DNut) and wing MNs (MNwm) have significant connectivity with hemilineages 6A, 7B, 2A, 19B, 12A and 3B". What is significant connectivity? Looking at the plot in Figure 8B, why is DNut -> 16B not considered significant? Is there a threshold and if so, what is the justification?

These plots aim to be descriptive rather than drawing hard quantitative thresholds between ‘significant’ and ‘non-significant’ connectivity. We have revised the text to remove the terms ‘restricted’ and ‘significant’ and to clarify our interpretation (L555-559).

In Figure 9G-H, this is a very interesting finding, but how do we know that the difference is real? Why not do a statistical test to compare the brain and VNC? Or create a null model network with edge swaps, etc. to compare against.

Statistical comparison between the brain and VNC may be problematic given differences in generating these connectomes, as well as missing connectivity (only half the brain is imaged) in the hemibrain connectome. Comparison to a null model is possible and for purposes of understanding motif frequency in general has already been done (see for example, Lin et al., 2024, Nature). However, a null or shuffled model is not required for comparing motif frequencies between brain or VNC neuropils as is the point of this particular graph. At present, we simply highlight a qualitative observation that will require future work to investigate.

Referring to Figure 12 in the main text, "we observe that the power MN upstream network is largely shared among all power MNs and is highly bilateral." Quantifying the fraction of shared upstream neurons from power MNs would make this statement much stronger. Particularly if compared to other non-power MNs. Or potentially using some other network comparison metric.

This is a good point. We have added cosine similarity to figure 6 for wing/haltere MNs to show the similarity between inputs across these MNs, and added text in section 2.3 (L461-465) and 2.5.3 discussing the cosine similarity (L987-988).

In Figure 13B, "Nearly 50% of these restricted neurons (totalling about 1200 per leg neuropil) have been serially matched across the six neuropils (Figure 13B)". There seems like a disconnect here. In the IR, CR, and BR columns, I see ~2750, ~500, and ~1250 neurons not in a serial set (~4500 total); I see ~1500, ~750, and ~1000 in a serial set (~3250 total). This would mean that ~58% of neurons are not in serial sets, ~42% are in serial sets. Shouldn't the conclusion be the opposite then? That surprisingly most intrinsic neurons are not repeated across leg neuropils. I find this fascinating if true. Perhaps there is some confusion on my part, however.

We now find that about half of the leg-restricted neurons are serially repeated across the 6 leg neuropil with similar morphology and connectivity, especially to the downstream leg motor neurons. Since first submission of this paper, we have identified some additional serial homologues while completing the systematic cell typing, described in the accompanying paper Marin et al. 2024. Figure 13B has now been updated to reflect this. In total, 3998 of 7684 restricted neurons (IR,CR,BR) have been assigned to a serial set or serial type. The sentence in the text has been adjusted to report that 52% of these restricted neurons are in serial sets (L1125).

In Figure 13D-E, "the Tect INs are not a homogenous population." Providing additional evidence could strengthen this statement. A connectivity matrix is shown in (D), followed by examples of morphologies in (E). What makes a population homogenous or heterogenous? For example, compared to all possible INs, the Tect IN morphology actually looks quite similar. Are those connectivity matrices in (D) really so different? What would a random selection of neurons look like?

Our sister paper, Marin et al. (2024), has looked into variation of connectivity across neurons of the entire VNC in much more detail, including clustering methods that include connectivity and other criteria for cell typing. Thus, we have now amended the text to direct the reader to that paper for more detail on variability of connectivity in the Tect INs, which were divided into 5 cell types in Marin et al. (2024) (L1027-1031). In addition, we have replaced our clustering by connectivity in Figure 13 with the cell type clusters from Marin et al. (2024).

In reference to Figure 13 - Supplement 1, "This standard leg connectome was very similar across legs, but there were small deviations 1051 between T1, T2, and T3 legs, as shown in Figure 13-Supplement 1." - what makes a deviation considered small? T1 seems to generally have many more synapses, T2 many less, and T3 a mixture depending on the connection. Also, are there lost connections or new connections? A quantification of these issues would be helpful instead of simply depicting the wiring diagrams.

The connections that differ are likely due to the reconstruction state of leg MNs. We have now stated this in the main text for clarification (L1143-1145). In the leg neuropils, T2 and T3 left hand side MNs have sparser dendritic arbors than the right hand side. Therefore the differences in Figure 13–Supplement 1, which are almost exclusively the connections between the leg restricted neurons onto leg MNs, seem stronger in T1. Future work, bolstered by additional datasets, will undoubtedly reveal further insight into the comparison of circuits for the different legs.

In Figure 15 - Supplement 2, "We used effective connectivity to identify leg DNs with similar MN connectivity patterns (Figure 15-Supplement 2). Of previously identified DNs, we found that DNg13 showed a highly similar effective connectivity fingerprint."

How was this similarity calculated? How do we know these particular DNs have similar effective connectivity? The connectivity matrix depicted is quite complex, with both layer and connectivity scores quantified at each location. A principled way of determining similarity would make this statement much stronger.

The similarity was calculated simply as the Euclidean distance between the effective connectivity matrix for each DN onto the set of MNs. While this is a straightforward comparison mathematically, effective connectivity calculations (as first introduced in this context by Li et al., 2020 by our collaborators Larry Abbott and Ashok Litwin-Kumar) have not yet been subject to functional validation. We therefore agree with the reviewer that this should not be over interpreted at this point. Future functional work should explore hypotheses suggested here and more quantitatively compare the similarity of different DN-MN pathways.

Minor notes:

In Figure 4E, the circles, squares, and triangles in the figure legend are too small. This is also true to some extent in the plot itself.

We have increased the size of the symbols in the legend and plot.

In Figure 8E right, the figure legend and x/y axes are not clear to me. Unfortunately, I'm not sure what the plot is showing because of this.

The right plot in figure 8E is the number of DN groups each MN group receives input from, at a threshold of 1% input. As this plot is redundant to the left plot, we have decided to remove it.

In Figure 8I, it would be interesting to see which neurons are directly downstream of DNs. One can't see layers 2/3/4 with the fan-out expansion of neurons and the y-axis scale.

We have revised the plot to better show cell composition of individual layers.

In Figure 19E, it would be helpful to also have a standard y-axis.

The panel has been revised accordingly.

**Reviewer #2 (Recommendations For The Authors):**

General:

In the Title, you do not mention DNs or MNs but these are a major focus of this study. The title could be more descriptive of the work.

Per the reviewer’s comments, we have revised the title to “Transforming descending input into motor output: An analysis of the *Drosophila* Male Adult Nerve Cord connectome”.

A glossary would be helpful, where all the paper's abbreviations and their definitions are provided in one place. Perhaps a hierarchical structure would help (for at least part of the glossary), so that terms like NTct, WTct, and HTct could be nested underneath UTct, for example.

We do include a glossary in the sister paper, Marin et al. (2024) and in this paper have included a short glossary in the first Figure. Please refer to these sources for abbreviation reference.

Introduction:

Define 'Premotor'.

We have defined ‘premotor circuits’ to be ‘circuits that directly or indirectly control motor output’ in lines 45-46.

It might be worthwhile to start with a broader introduction sentence than the current one that focuses just on the fly, in order to emphasize the impact of MANC as the first complete connectome of a motor circuit in any animal with limbs or wings.

We have revised the introductory paragraph per the reviewer’s suggestions.

"Muscles in the leg are not innervated uniformly; indeed, in the T1 legs the number of MNs per muscle varies by as much as an order of magnitude" needs to specify the axis of variability more clearly - the authors probably mean variability across muscles in the leg (not variability across individuals for example) but I think the current sentence is a bit ambiguous in that respect.

We have reworded this sentence to clarify this point (L132-133).

Line 182 end of paragraph: It would be useful to point out explicitly what makes the MANC project valuable in the context of a similar FANC project - for example, that the MANC connectome is more complete, is a male (so interesting for anyone interested in sexual dimorphism), and gives the field an n=2 for VNC connectome datasets.

We agree, and have added a sentence describing the benefits of the MANC connectome on L209-212.

Line 213: A brief phrase or sentence of context could be provided to help unaware readers understand that 42% of synaptic connectivity being captured is in the same sort of range as previous datasets like the hemibrain and likely leads to the vast majority of important cell-cell connections being identified (perhaps cite Buhmann et al 2021 Nature Methods which does an analysis of this), and therefore is a reason to think highly of this dataset's quality and its potential for impact on the field. The sentence at the end of this paragraph doesn't quite do it for me.

We have added the comparison of MANC synapse completeness to that of the Hemibrain, and revised the ending sentence in L234-237.

Line 271: Clarify what happened to the remaining 15% of DNs that weren't able to be assigned to a tract. They travelled outside the tracts, or data quality issues prevented assignment, or something else?

Indeed, some DNs could not be assigned to a tract as they traveled outside of all axon tracts and did not bundle with other DNs. We have added this explanation to the text (L300-301).

Figure 1:

The pie chart "DN postsynaptic partners by neuron class" is a bit hard to interpret without having another pie chart next to it showing "Neurons in MANC by neuron class". I know these numbers are written on the schematic but it would be nice to be able to easily tell which cell classes are overrepresented or underrepresented in the set of postsynaptic partners of DNs. e.g. It's obvious that ANs are overrepresented and DNs are underrepresented in the set of postsynaptic partners of DNs, but it would be nice if readers didn't have to do any mental math to figure out if INs or MNs are under/overrepresented.

We agree and have added a pie chart of the neuron class composition of the entire VNC to Figure 1.

"35.9% of leg MNs are matched to FANC" Why is this number so low? Because FANC motor neurons were only identified in T1, so the remaining 2/3rds of leg MNs in MANC weren't matched? How successful was matching for the neurons where it was actually attempted?

For this work, we only matched the T1 neurons across the two datasets. This was both a way of checking that we found everything in these segments and a way of being more sure of muscle target assignments as our collaborators in the FANC dataset had generated extensive light level data to match motor neurons with their target leg muscles. The T2 and T3 MNs were not fully proofread or identified in FANC, precluding further analysis, and leading to the 35.9% matched number. We hope to be able to compare between these datasets more thoroughly in future, and have matched all the premotor leg restricted intrinsic neurons of our standard connectome to FANC. We report on their stereotypy in our latest preprint, Stürner, Brooks et al. 2024.

Figure 2:

Figure 2A: Perhaps darken the color of the MTD-III skeletons. Currently, they're so light it's hard to see, and this is one of the most interesting tracts because the claim is that it's a new tract.

We take the reviewer’s point, however, the color scheme used for the tracts in Figure 2 is coordinated between multiple figures and figure panels, and thus we would prefer to keep it as is. If readers would like to examine DNs of a particular tract, we encourage them to retrieve said DNs using the tract annotations in NeuPrint.

Figure 2 supplement 1: It's not clear to me what I should be getting out of seeing the right side DNs as well. If you want readers to be able to visually compare the left and right side morphologies and appreciate the high degree of symmetry, you may want to put the left and right side DN panels side-by-side. Perhaps do that (show both the left and right side DNs) for one or two tracts in the main Fig2, and then leave out the remaining panels - or if you want to include the remaining panels, explain more clearly what readers are supposed to learn from seeing them.

We agree and have now removed Figure 2 supplement 1.

Figure 2C caption: Instead of "DN primary neurites" I think the authors probably mean "longest single branch of each DN" or something along those lines. I think "primary neurite" is usually used to refer to the thick non-synaptic branch coming out of a neuron's soma, which can't be how it's being used here.

We agree and have changed all references to ‘primary neurite’ for DNs to ‘longest neurite’.

Figure 2D+E: Perhaps add an overall % of neurons of each class to the legend. I ask because I would be very interested to know what % of all DNs exist as single pairs versus as populations, and I imagine that could be a number that is quoted a fair amount by others in the field when talking about DNs.

We agree and have added the overall percentage of each neuron class to the results (L275-276) and Figure 2 legend.

Figure 3:

UTct.IntTct neurons are by far the largest class of DNxn neurons, so would it be worth calling these the DNxt class (DN projecting to some combination of tectulum neuropils), to mirror the DNxl class? I would vote for doing that.

Thanks for the suggestion. However, the subclass naming scheme for DNs had been coordinated between multiple groups of people working on MANC reconstruction and annotation. As making changes to subclasses will impact many analyses that have already been completed for existing work, we will refrain from doing so.

Figure 3G feels a bit out of place in this figure and under-explained

We have clarified in the text our citations to Figure 3G to better explain our interpretation of this data.

Figure 4

"DNp20 has few vesicles and may be electrically coupled": If I'm correct that DNp20 is also known as DNOVS1 and is the second largest diameter axon in the neck after the giant fiber, then yes, Suver et al. 2016 J Neurosci show that this DN is gap junction coupled to neck motor neurons (see their Fig 2F). This neuron (along with the giant fiber) is enough of an outlier that it might be more representative to show a different, more canonical DN that has a low prediction probability.

The reviewer is right that DNp20 is also known as DNOVS1 with known gap junction coupling. We now clarify in the text (L366) how we think that could lead to a lower neurotransmitter prediction score, which is what we were trying to illustrate.

Figure 4E: It looks like only a single DN has more inputs (~11000) than outputs (~9000), is that right? It could be interesting to dedicate some panels and text to the connectivity profile of that one unique neuron.

Yes, that is correct, there is just one pair of DNs, DNxn166, that receives more input than it gives output (the two triangles lie on top of each other). We think that the other DN pair in that same box (more variable in total synapse number and therefore the triangles are further apart) also receives an unusually high amount of input versus output. The morphology of these two types are shown in Figure 4F and they both have fine processes that look more like dendrites, especially when compared to other DNs such as the ones in 4G. Unfortunately, neither of these two types have been matched to light microscopy images so we cannot say if they have the same type of morphology in the brain, or further explore their brain connectivity, at this time point.

Figure 4E: "black rectangle ... gray rectangle" don't look different shades to me. It's obvious which is which based on where they are in the graph but if you want to color code this, pick more separate colors. Or code it with something other than colors.

We have made the rectangle in Figure 4E a lighter shade of grey and added labels to refer to the panels D, F and G. The figure legend now also describes more clearly that we are plotting every DN as a single shape and exactly how many DN types are included in those rectangles to avoid confusion.

Figure 5:

"subclass is their two-letter muscle anatomical category" should be explained better, I'm not sure what "muscle anatomical category" means.

We have changed the wording in the Figure 5 legend to better clarify that MN subclasses are the broad muscle category that they innervate (e.g. legs, wings).

Figure 7:

Leg MN identification and serial homology.

Why are there no tarsus reductor (tarm1 and tarm2) motor neurons? Do we not know their anatomy from light microscopy well enough, perhaps? Were these MNs identified in FANC? Is it reasonable to guess that the remaining small number of unidentified T1 leg motor neurons in MANC would control these muscles? I think Marta Moita's lab has some ongoing projects on these muscles (see Twitter), so if more LM data is needed perhaps it will come from them.

We now know that the small number of unidentified T1 leg motor neurons (a T1 pair with a serial T2 pair, serial set 17664) are not in fact MNs. A new and unpublished dataset (Janelia whole male CNS volume, the optic lobe from which has been published as Nern et al., 2025) shows they have axons within the VNC. The MN annotation for these neurons has been removed and they now have the type name INXXX471. Thus, we have no T1 leg MNs without a muscle target annotated. Our muscle target annotation comes from matching to the FANC dataset that has also not annotated tarsus reductor MNs. We suspect that the tarsus reductor MNs are hard to distinguish from the tarsus depressor MNs of which there are 5 per side and segment.

It seems there are a few more leg motor neurons in MANC vs FANC. Any indication of which muscles they control?

See above.

-Figure 7E: A qualitative comparison between the cosine similarity results here and from FANC could be useful. What generally is the same versus different? Any indication of male/female differences?

We observe no differences in the cosine similarity of T1 leg MNs between MANC and FANC and only very minor differences between T1, T2 and T3, as shown in Figure 7. In our most recent work, now on bioRxiv (Stürner, Brooks et al., 2024), we were able to find all intrinsic leg serial sets that we included in our standard leg premotor circuit here in the FANC dataset. We do not see any differences between them in terms of morphology, and while we have several cases in which we are still missing 1 of the 6 neurons in a serial set in FANC, we see similar connectivity when comparing small circuits. We have also found almost all neurons interconnecting the legs, with some very interesting exceptions, mainly coming from the abdomen, that we believe are male specific. These male-specific neurons can also be found in this preprint (Stürner, Brooks et al., 2024).

Figure 8

Figure 8A: Why are ~1/3rd of the wing and leg motor neurons considered populations instead of pairs? I thought essentially all wing and leg motor neurons have unique morphologies.

Pair vs populations are assigned based on MN morphology and connectivity. For the wing MNs, many sets of DVMns and DLMns have near-identical morphology and connectivity, are not easily distinguishable in the VNC and are categorized as a ‘population’. For the leg MNs, there are ‘true’ population MN types that provide multiple innervation of the same muscle.

The text states "up to a maximum of 20% [traversal probability] (corresponding to a synapse input fraction of 1)" but I interpret the bottom of Figure 8G to have flipped values, where a synapse input fraction of 0.2 yields a traversal probability of 1. Is there a mistake here or have I misunderstood?

Thank you for pointing this discrepancy out. The text description was indeed flipped, and we have corrected this error.

Caption for J says "Layers without neurons are omitted". How is it possible to have a layer without neurons?? Something about how the traversal is done doesn't seem to be explained clearly enough. If it's really possible to have a layer without neurons, I think the approach might need to be revisited as this seems quite strange.

Here, ‘layer’ should be viewed as a nonlinear measure of indirect connectivity combining path length and synaptic weights. Layers without neurons are possible due to the details of the calculation–layer position is assigned probabilistically by the downstream synapse connectivity of the source neurons, and the probability is scaled up to 1 at an input synapse fraction of 0.2. Neuron-to-neuron connectivity of an input synapse fraction of > = 0.2 is very rare in the VNC connectome and thus neurons strictly assigned to layer 2 downstream of each DN type are similarly rare. We have updated the figure legend for figure 8 to better explain this.

Section 2.6

"flies have been shown to walk normally without proprioceptive feedback, suggesting that inter- and intra-leg coordination is not strictly dependent on sensory feedback loops from the legs" is quite a drastic overinterpretation of that paper's results. The ablation there was not complete (some subtypes of sensory neurons were not perturbed), and the perturbed flies certainly walked with some defects. This statement certainly should be removed or significantly softened.

Thank you for pointing this detail out. The term ‘normally’ has been removed from this sentence to soften the statement.

Figure 13, Standard leg connectome

Unfortunately, the motor neurons controlling the tarsus could not be included here, I suppose due to the difficulty in identifying the T2 and T3 homologs for these motor neurons. This should be mentioned in the text. This version of the standard leg connectome is without a doubt still an incredibly valuable discovery, but readers should be made aware that this version of the standard leg connectome does in fact lack the motor neurons for one joint.

The MNs controlling the tarsus could not be matched with high confidence. We have added a sentence pointing this out when the leg circuit is introduced (L1141-1142).

The focus here is on locomotion is the absence of other behaviors whereas the legs are responsible for grooming, reaching, boxing, etc. How should we consider the leg connectome in light of this?

This is a very good point, and we have indeed found known grooming neurons that target our leg premotor circuit (L1158-1161). We’ve now added this observation to the Discussion (L1949-1951).

Minor points

L84 - re: Descending neurons work together - cite Braun et al., bioRxiv 2023; cite Yang HH bioRxiv 2023 .

We agree that these papers are relevant to the function of DNs in combination, and have added them to the introduction (L83-84, 86-87).

L193 - "intrepid" is overly florid language; similar for L1507 "enigmatic".

We have replaced these words with suitable synonyms.

L273 - The acronym "ITD" is not explained. Please check all other acronyms. Related, it would be good to include a Table or Box with all acronyms for the reader.

We have added the full name of the ITD to the text. A glossary is available in Figure 1, and a full glossary of MANC terms is available in Table 1 of our sister paper, Marin et al. 2024.

-L514, you state that hemilineages 6A and 6B unexpectedly produce uncoordinated leg movements (flight-related was expected). However, Harris didn't study animals in tethered flight but headless on the ground.

The experimental setup of Harris et al. was capable of assessing flight-like motor output even if not true flight, as seen in the predominantly wing movement phenotypes of activating hemilineages 7B, 11A/B and 2A. We now also note that hemilineage annotation in Marin et al., 2024, shows that the 6B hemilineage has some projections into the leg neuropils, in support of a leg motor role in addition to an upper tectular role (L570-571).

L1425 - "the TTM" is repeated twice.

This sentence addresses both the TTM and its MN (TTMn). We have revised this sentence to improve clarity by expanding the full name of TTM in that paragraph and leaving TTMn abbreviated

L1728 - Ascending neuron projections to the brain - cite Chen et al., Nat Neuro 2023.

We agree that Chen et al. 2023 is relevant to the discussion of AN function, and have added this citation (L1836-1838).

L1817, It is a good idea to compare with previous predictions for circuit control. But these originate from non-*Drosophila* work as well. Please cite and consider the original models from Buschges, Cruse, Holmes, and others.

Thanks for the suggestion. We now cite the non-*Drosophila* literature as well. (L1971)

L1827, how precisely should these "theories" be updated? Be explicit.

We summarize in the sentences before what is different in comparison to one of the suggested models. We have now additionally added examples to the sentence (L1942-1945) to suggest that theoretical leg circuits need to account for the posterior-to-anterior as well as anterior-to-posterior connections between leg neuropils, as well as relative lack of connectivity between the left and right mesothoracic leg neuropils.

L1831, include a discussion about another alternative which is through mechanical coupling and sensory feedback.

We agree that leg sensory input likely contributes to leg locomotor circuits. We have added the following sentence to point out that annotations of sensory neurons in MANC are available through work in a companion paper (Marin et al. 2024), and future work is necessary to examine the contribution of sensory input to leg motor circuits (L1954-1956).

Methods

https://flyconnectome.github.io/malevnc/ link doesn't work.

We have updated the link.
